# Molecular Identification and Antimicrobial Resistance Characteristics of Extended-Spectrum Beta-Lactamase Producing *Klebsiella pneumoniae* Isolated from Captive Wild and Migratory Birds

**DOI:** 10.3390/vetsci12060556

**Published:** 2025-06-06

**Authors:** Muhammad Mujahidul Islam, Md Bashir Uddin, Hemayet Hossain, Milton Roy, Ruhena Begum, Piash Kumer Ghosh, Md. Mahfujur Rahman, Ho-Seong Cho, Md. Mukter Hossain

**Affiliations:** 1 Department of Medicine, Sylhet Agricultural University, Sylhet 3100, Bangladesh; mujahid.sau18@gmail.com (M.M.I.); bashir.vetmed@sau.ac.bd (M.B.U.); miltonroy.vet@student.sau.ac.bd (M.R.); dr.ruhenabegum@gmail.com (R.B.); piash.vabs@student.sau.ac.bd (P.K.G.); mahfuj.vetmed@sau.ac.bd (M.M.R.); 2 Department of Anatomy and Histology, Sylhet Agricultural University, Sylhet 3100, Bangladesh; hemayet.vabs@student.sau.ac.bd; 3 College of Veterinary Medicine and Bio-Safety Research Institute, Jeonbuk National University, Iksan 54596, Republic of Korea

**Keywords:** *Klebsiella pneumoniae*, molecular identification, extended-spectrum *β*-lactamase, multidrug resistance, migratory and wild birds

## Abstract

The emergence and dissemination of antibiotic-resistant *Klebsiella pneumoniae*, particularly those are extended-spectrum beta-lactamase (ESBL) producers, thought to pose a serious threat to global health. This study aimed to isolate and identify the ESBL-producing *K. pneumoniae* from captive wild and migratory birds in Bangladesh along with their antimicrobial resistance characteristics. In this investigation, standard bacteriological methods were used to detect *K. pneumoniae* in 219 fecal samples. The positive isolates were confirmed by PCR and antibiotic susceptibility testing was performed by disk diffusion method. *K. pneumoniae* was detected in 93 (42.47%, 95% CI: 35.8–49.3) out of 219 fecal samples. The prevalence of *K. pneumoniae* was higher in captive wild birds (50%; 40/80) compared to migratory birds (38.1%; 53/139). The isolates showed high resistance to ampicillin (69.9%) and streptomycin (64.5%). Conversely, the highest sensitivity was recorded for trimethoprim-sulfamethoxazole (84.95%), followed by levofloxacin (79.57%) and gentamicin (69.89%). Molecular screening revealed that all positive isolates harbored *bla*_TEM-1&2_ encoding genes, with 45.2% and 15.1% carried *bla*_SHV-1_ and *bla*_OXA-1,4&30,_ respectively. Additionally, resistance genes ***strA*** (30.1%), ***tetA*** (9.7%), and ***sul1*** (9.7%) were detected. The Multiple Antibiotic Resistance (MAR) index ranged from 0.18 to 0.64, with 63.4% of isolates classified as MDR. The isolation of MDR and ESBL producing *K. pneumoniae* from captive wild and migratory birds suggests that these birds may serve as reservoirs for the spread of these bacteria, potentially impacting public health in the study region.

## Simple Summary

This study investigated the molecular identification and antimicrobial resistance characteristics of *Klebsiella pneumoniae* producing extended-spectrum beta-lactamase (ESBL) in captive wild and migratory birds in northeastern Bangladesh. Out of 219 fecal samples, 42.47% tested positive for *K. pneumoniae*, with a higher prevalence observed in captive birds. The isolates showed significant resistance to ampicillin and streptomycin, while trimethoprim-sulfamethoxazole and levofloxacin were the most effective antibiotics. PCR detected key resistance genes, including *bla*_TEM-1&2_ (100%), *bla*_SHV-1_ (45.2%), and *bla*_OXA-1,4&30_, along with *strA*, *tetA*, and *sul1*. The multiple antibiotic resistance (MAR) index varied from 0.18 to 0.64, with 63.4% of isolates classified as multidrug-resistant (MDR). These findings suggest that wild and migratory birds may serve as reservoirs and disseminators of MDR and ESBL-producing *K. pneumoniae*, posing a potential threat to public and animal health. Continuous surveillance and a One Health approach are recommended.

## 1. Introduction

Wild and migratory birds represent a large proportion of the estimated 10,000 bird species globally [1]. Migratory and wild birds are a potential reservoir and are able to propagate zoonotic pathogens and resistant bacteria [2]. These birds frequently travel across countries and continents, facilitating the spread of infectious agents through fecal contamination in water bodies such as lakes, ponds, wetlands, haor etc. [3,4]. Through migration, the birds may acquire or disseminate antimicrobial resistant (AMR) and multidrug resistant (MDR) bacteria [4,5]. Notably, fecal shedding by birds can introduce pathogens into shared environments, which may subsequently infect humans, domestic animals, or wildlife [6]. This risk is particularly relevant in regions like Bangladesh, where seasonal migratory bird influxes are common and many communities rely on open water sources for daily activities [7,8].

*Klebsiella pneumoniae (K. pneumoniae)* belongs to the family *Enterobacteriaceae*. Enterobacteria are bacteria that live in the intestines of mammals and some birds, and they can spread throughout the environment and become ubiquitous if the right conditions exist [9]. Because they are found in intestine of wild and migratory birds, they have great significance in animal and human health. *K. pneumoniae* is a common Gram-negative bacterium recognized as a primary pathogen, often associated with respiratory diseases in birds [10]. It may be isolated from stool samples and the oropharynx of various species of healthy parrots and passerines [10,11,12]. It frequently acts as a respiratory pathogen, particularly among immunosuppressed and stressed birds [13]. The microbiota of caged birds is likely to change as a result of their management, favoring Gram-negative bacteria colonization [14]. In certain cases, *K. pneumoniae* can lead to encephalitis, lung infections, and renal failure. Localized infections of the skin, lips, upper respiratory tract, and crops, specifically in psittacine birds, are more prevalent [15].

*K. pneumoniae* is one of the most common producers of extended-spectrum β-lactamases (ESBLs), along with *Escherichia coli*. Several virulence factors, including a polysaccharide capsule, muco-viscous exopolysaccharides, lipopolysaccharides (LPS), adhesins, and iron acquisition systems have the capacity to acquire antimicrobial resistance determinants, making *K. pneumoniae* particularly dangerous [16]. ESBL-producing strains are particularly concerning due to their resistance to β-lactam antibiotics and carbapenems genes, leaving limited treatment options such as imipenem or meropenem [17,18]. The emergence of ESBL- and carbapenemase-producing *K. pneumoniae* is a critical priority pathogen for global health intervention due to its role in hospital-acquired infections and its limited therapeutic options [19,20,21].

Despite its clinical relevance, the role of wild and migratory birds as reservoirs of ESBL-producing *K. pneumoniae* in South Asia, particularly Bangladesh, remains poorly understood. Recent surveillance studies from other regions (e.g., China, Europe, and Egypt) have revealed varying prevalence rates of *K. pneumoniae* in avian populations, ranging from 1.5% to over 50% [4,18,22]. However, molecular data from avian hosts in Bangladesh are lacking. Therefore, the present study aimed to isolate and characterize *K. pneumoniae* from fecal samples of captive wild and migratory birds in northeastern Bangladesh. Specifically, the study focused on identifying the prevalence of ESBL-producing strains, profiling antimicrobial resistance patterns, and detecting key resistance genes using molecular techniques.

## 2. Materials and Methods

### 2.1. Study Area and Species Selection

**Figure 1:**
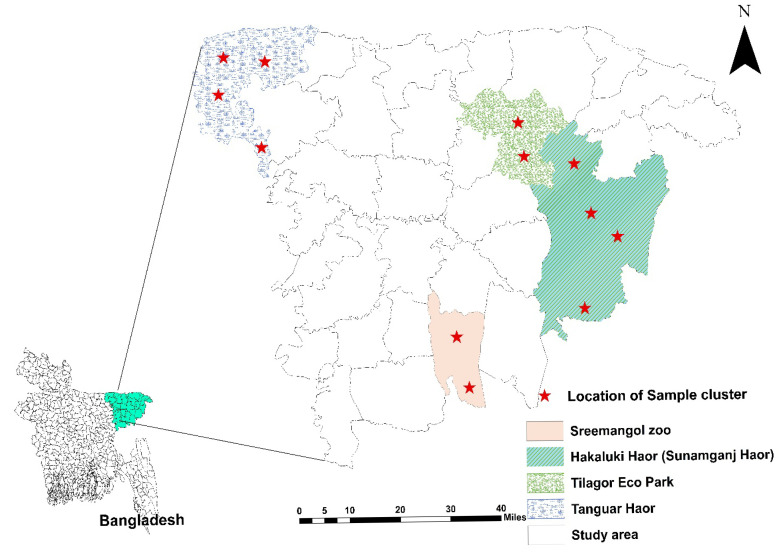
Study area map represents the location of sample collection. The figure was generated using ArcMap 10.8.

From January 2022 to July 2022, a cross-sectional investigation was conducted in Haripur, the Hakaluki Haor of Moulvibazar, the Tanguar Haor, and parts of the Tahirpur upazila of Sunamganj District, based on the accessibility and density of birds (Figure 1). Feces from captive wild birds were obtained from Tilagor Eco-Park, Sylhet, and the Bangladesh Bannyaprani Sheba Foundation, Sreemangal, Bangladesh. Migratory bird species were prioritized due to their potential role in the long-distance dissemination of pathogenic bacteria. Captive wild birds were also included to provide a baseline for AMR prevalence in local avian populations. Species were selected based on prior studies indicating their exposure to anthropogenic influences in agricultural areas, wetlands, or rural feeders.

### 2.2. Sample Collection

In all, 219 fecal samples were gathered from various sites in Sylhet region, Bangladesh. These locations were chosen because they are home to a variety of species of migrating and wild birds in captivity. A sterile swab stick was used to aseptically collect feces samples. The feces were then placed in sterile containers containing buffered peptone water (BPW) (Hi media, India) at 1:10 dilution, and were labeled accordingly. For consistency, all samples were processed within 24 h to ensure data reliability.

### 2.3. Isolation and Identification of K. pneumoniae

The samples from BPW were cultured on MacConkey Agar (MCA; Oxoid, Southampton, UK) and then on Eosin Methylene Blue Agar (EMBA; Oxoid, UK), and by streaking and incubation at 37 °C for 18–24 h. Culture positive samples were sub-cultured several times to obtain a pure culture. The growth of large, mucoid, pink, or pink to purple colonies on EMB and MacConkey agar plates demonstrated the growth of *K. pneumoniae*. Gram’s staining method and various biochemical tests were performed to screen the single pure colonies for additional confirmation. These tests included catalase, sugar fermentation, methyl red, Voges–Proskauer, and citrate utilization (Supplementary Materials). Pure cultures were stored in two separate Eppendorf tubes and preserved in Brain Heart Infusion broth (BHIB; Oxoid, UK) with 15% glycerol supplement, and stored at −40 °C until further use.

### 2.4. Genomic DNA Extraction

The DNA extraction was carried out according to the manufacturer’s instructions (AddBio Inc. Ltd., Dajeon, Republic of Korea). Briefly, 200 μL of cultivated cells were extracted overnight and centrifuged at 13,000× *g* rpm for 30 s. The supernatant was disposed of and 200 μL of lysis solution was pipetted in and suspended. After adding 20 mg/mL of proteinase K solution, the mixture was incubated at 56 °C to ensure full lysis. The mixture was thoroughly homogenized and incubated at 56 °C for 10 min following centrifugation and the addition of binding solution. Following the addition of 100% ethanol, the lysate was cautiously moved to a spin column tube, centrifuged, and then cleaned out using washing solutions. The spin column was again centrifuged at 13,000× *g* rpm for 1 min in order to remove any last traces of ethanol. Lastly, 30 μL of elution buffer was added to the spin column and was allowed to sit for 1 min at room temperature. The genomic DNA was eluted by centrifugation and kept at −20 °C for further examination. The quantity and purity of extracted DNA were verified using a NanoDrop spectrophotometer.

### 2.5. Molecular Detection of K. pneumoniae by PCR

Species-specific primers were used for the amplification of the *rpoB* gene of *K. pneumoniae* (F-CAACGGTGTGGTTACTGACG and R-TCTACGAAGTGGCCGTTTTC). Each PCR reaction contained 25 µL reaction volume with 12.5 µL of 2× master mix, 2 µL of primer mix, and 5 µL of a DNA sample. The 25 µL volume was adjusted by adding 5.5 µL of nuclease-free water appropriate for high-performance liquid chromatography. PCR was performed using a thermal cycler (DLAB Scientific Inc., Alhambra, CA, USA) with the following cycling conditions: initial denaturation at 95 °C for 5 min, followed by 35 cycles of denaturation at 95 °C for 1 min, annealing at 55 °C for 1 min, and extension at 72 °C for 2 min, with a final extension at 72 °C for 10 min. After that, the PCR product was visualized by electrophoresis, revealing a unique band of 108 bp for *K. pneumoniae (*Figure S1A). A 100 bp ladder (AddBio Inc., Dajeon, Republic of Korea) was included to assist with PCR product size estimation.

### 2.6. Double-Disk Synergy Test (DDST) for ESBL Detection

The phenotypic detection of ESBL production in K. pneumoniae isolates was performed using the Double-Disk Synergy Test (DDST) [23]. A bacterial suspension equivalent to a 0.5 McFarland standard was prepared from each isolate and evenly inoculated onto Mueller–Hinton agar plates using a sterile cotton swab. An amoxicillin-clavulanic acid disk (30 µg) was placed at the center of the plate, and cefotaxime (30 µg) and ceftriaxone (30 µg) disks were positioned 20 mm apart (center-to-center) on either side of the central disk. Plates were incubated at 37 °C for 24 h.

Following incubation, plates were examined for the presence of a characteristic “keyhole” or synergy zone between the cephalosporin disks and the amoxicillin-clavulanate disk. The appearance of this zone indicated the inhibition of ESBL activity by clavulanate, confirming ESBL production.

### 2.7. Antimicrobial Susceptibility Testing

The agar diffusion method developed by Kirby–Bauer was employed to assess antibiotic sensitivity. The antibiotics that have been utilized come in disk form. Three categories (sensitive, intermediate, and resistant) were established from the clear zone that developed during this test. Using a tube, *K. pneumoniae* isolates were extracted from one or two pure colonies and mixed in physiological NaCl. The turbidity of the solution was assessed using the McFarland 0.5 standard. A volume of 0.2 mL was then obtained and gently rubbed across the Mueller–Hinton agar (MHA: Oxoid, UK) medium. Eleven antibiotics (Oxoid, UK) were used for sensitivity testing (Table S1). Findings were ascertained after 18–24 h incubation at 37 °C. Using a manual millimeter scale, after measuring the diameter of the zone of inhibition encircling the discs, the findings were contrasted with the CLSI breakpoints [24].

### 2.8. Identification of ß-lactamase Encoding Genes

Reference primers were used to detect ß-lactamase genes (Table 1). All *Klebsiella pneumoniae* isolates underwent PCR screening to identify the ß-lactamase genes, including *bla*_TEM-1&2_, *bla*_SHV-1_, and *bla*_OXA-1,4 &30_ (Figure S1B–E). Thermal cycle conditions are described in the Supplementary Materials.

### 2.9. Detection of AMR Genes

The targeted AMR genes *tetA* and *strA* were amplified using multiplex PCR using two sets of particular primer pairs (Table 1). Every PCR reaction had a volume of 25 μL and included 5 μL of DNA template from PCR positive isolates, 12.5 μL of 2× master mix (Add Bio Inc., Daejeon, Republic of Korea), 0.5 μL of each primer (10 pmol/L concentration) for forward and reverse, and 5.5 μL of nuclease-free water. The *sul1* genes were found using a different uniplex PCR test. To perform the PCR assay, a combination of 25 μL reaction volume was used, which included 5 μL of DNA template obtained from isolates of *K. pneumoniae*, 12.5 μL of 2× master mix (Add Bio Inc., Dajeon, Republic of Korea), 1 μL each of the forward and reverse primers (10 pmol/L concentration), and 5.5 μL of nuclease-free water. Thermal cycle conditions are described in the Supplementary Materials.

### 2.10. MAR Index and MDR

Using the formula MAR = (the number of antibiotics to which an isolate was resistant)/(the total number of antibiotics tested), the MAR index was computed in accordance with the parameters as previously established [28]. When an isolate showed resistance to at least three different classes of antibiotics, it was classified as multidrug resistant (MDR). The MAR index ranged from 0 to 1, where values close to 1 indicated strong resistance and values around zero indicated high sensitivity. A significant level of resistance or high-risk bacterial contamination was indicated by an index of 0.20 or above.

### 2.11. Statistical Analysis

The information was assimilated, categorized, and structured into Excel spreadsheets. The prevalence for different diseases was calculated using following formula [29,30]:

$$\mathrm{Prevalence}=\frac{Number\;of\;current\;cases\left(new\;and\;preexisting\right)at\;a\;specified\;point\;in\;time}{Population\;at\;the\;same\;specified\;point\;in\;time}\times 100$$

Chi-square (χ^2^) analysis was done to evaluate the relationships between the different explanatory factors. Fisher’s Exact Test was used when the predicted count in a cell was less than five and it happened in at least 20% of the cells [31]. Spearman correlation coefficient was performed using package R (RStudio 4.3.3). The data analysis was conducted with SPSS version 26. The threshold of significance was defined as *p* < 0.05. The study area map was created using ArcMap 10.8 and the other plot was created using GraphPad Prism 8.4.

## 3. Results

### 3.1. Prevalence and Distribution of K. pneumoniae

**Figure 2:**
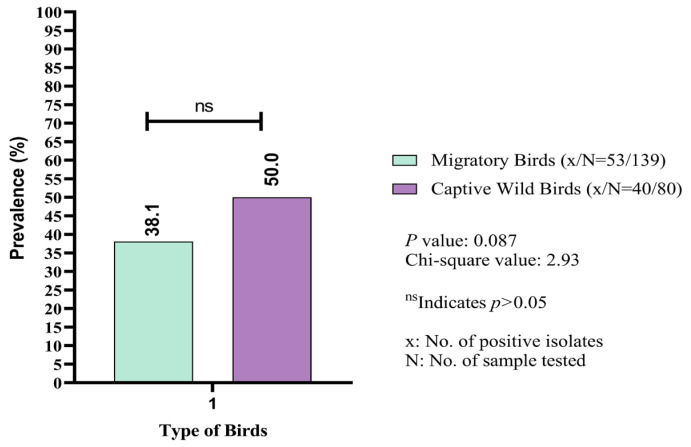
Comparative assessment of prevalence (%) of *Klebsiella pneumoniae* between migratory and captive wild birds.

The overall prevalence of *K. pneumoniae* isolated from both migratory and captive wild birds was 42.5% (93/219; 95% CI: 35.8–49.3) (Table 2). The number of positive isolates varied significantly (*p* < 0.05) across different geographical locations in Sylhet Division. The highest prevalence of *K. pneumoniae* was recorded in Haripur (63.2%; 12/19). At Tilagor Eco-Park, only 25% (9/36) of the fecal samples from captive wild birds were positive for *K. pneumoniae*. Similar prevalence rates were observed in the Sunamganj Haor (43.6%) and the Sreemangal Zoo (44.3%) (Table 2). The frequency rate of *K. pneumoniae* isolated from captive wild birds was also observed (Table 3). Notably, the prevalence of *K. pneumoniae* was higher in fecal samples from captive wild birds (50%; 40/80) compared to migratory birds (38.1%; 53/139) (Figure 2).

### 3.2. Antibiogram Profile

**Figure 3:**
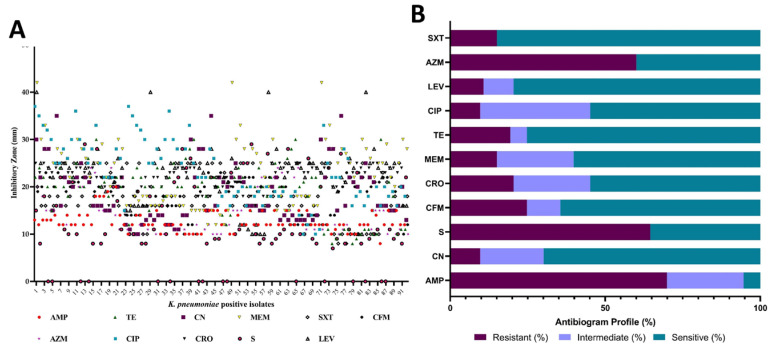
Zone of inhibition (mm) of 11 antimicrobials against positive isolates of *K. pneumoniae* (**A**). AMP: ampicillin, TE: tetracycline, CN: gentamicin, MEM: meropenem, SXT: trimethoprim-sulfamethoxazole, CFM: cefixime, AZM: azithromycin, CIP: ciprofloxacin, CRO: ceftriaxone, S: streptomycin, LEV: levofloxacin. The antibiogram profile showing the percent (%) of sensitive, intermediate, and resistance of the tested antimicrobials (**B**).

Most antimicrobials showed a minimum zone of inhibition (>10 mm) against the isolated *K. pneumoniae,* except for streptomycin. The overall zone of inhibition ranged between 15–25 mm for most positive isolates (Figure 3A). Streptomycin exhibited no inhibitory activity against 16 isolates. Among the selected antibiotics, the highest resistance was observed with ampicillin (69.89%), followed by streptomycin (64.5%). Conversely, the highest sensitivity was recorded for trimethoprim-sulfamethoxazole (84.95%), followed by levofloxacin (79.57%) and gentamicin (69.89%). Almost all the selected antimicrobials demonstrated sensitivity to some extent against the isolated pathogens (Figure 3B and Table S2).

### 3.3. MAR and MDR

The MAR index for multiple antibiotic resistance values varied from 0.18 (resistant to at least two antibiotics) to 0.64, with an average of 0.33. One isolate was resistant to eight out of 11 antimicrobials, and four isolates were resistant to seven antimicrobial agents, with an average MAR index of 0.54. Overall, 59 isolates (63.4%) out of 93 were resistant to more than three classes of antimicrobials (MDR).

### 3.4. ß-lactamase Encoding and Antimicrobial Resistance Gene Frequency

**Figure 4:**
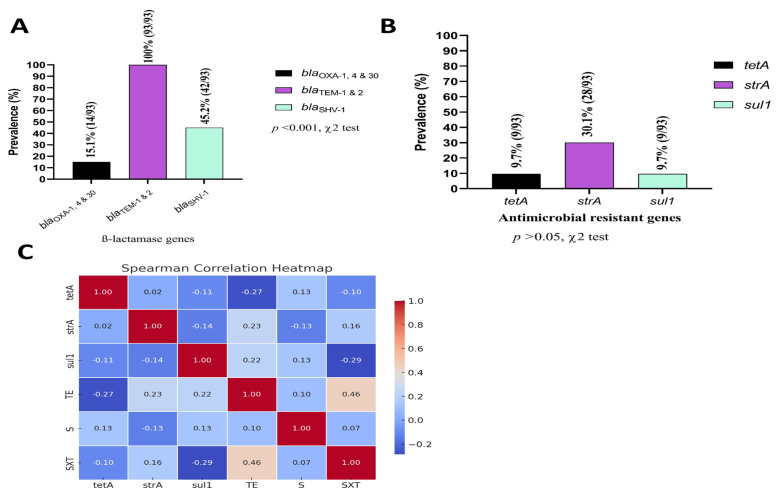
Frequency (%) of *ß*-lactamase encoding genes (**A**); Frequency (%) of selected antimicrobial resistant genes (**B**); Spearman correlation coefficient (phenotype–genotype correlation) among the most common antibiotics used against *K. pneumoniae* in this region with their respective genes (**C**). TE: tetracycline, SXT: trimethoprim-sulfamethoxazole, S: streptomycin.

All *K. pneumoniae* positive isolates harbored *bla*_TEM-1&2_ broad spectrum ß-lactamase encoding gene, while only 14 isolates (15.1%) carried *bla*_OXA-1,4&30_. Additionally, *bla*_SHV-1_ was detected in 42 (45.2%) of the 93 positive samples (Figure 4A). This study screened for three common antimicrobial resistance genes in captive wild and migratory birds, finding that 28 (30.1%) isolates were positive for *strA* and nine (9.7%) isolates were positive for *tetA*, and *sul1* (Figure 4B).

### 3.5. Phenotype–Genotype Correlations

The Spearman correlation analysis identified several key findings regarding the relationships between genotypes and antibiotic susceptibility test (AST) phenotypes (Figure 4C). The tetracycline resistant gene (*tetA*) exhibited a moderate negative correlation with tetracycline (TE) (r_s_ = −0.27), while showing a weak positive correlation with streptomycin (S) (r_s_ = 0.13). *strA* demonstrated a moderate positive correlation with TE (r_s_ = 0.23), indicating a potential link between the gene and TE resistance, along with a weak positive correlation with trimethoprim-sulfamethoxazole (SXT) (r_s_ = 0.16). Similarly, *sul1* presented a weak positive correlation with TE (r_s_ = 0.22) and a negative correlation with SXT (r_s_ = −0.29). Overall, the analysis revealed mostly weak correlations between the genotypes and the AST phenotypes, with a few moderate associations observed for specific gene–antibiotic combinations.

### 3.6. Association Between Resistance Genes and Phenotypic Antibiotic Resistance

The association between genotypic resistance markers and corresponding phenotypic antibiotic resistance was evaluated using binary logistic regression analysis (Table 4). The analysis showed that the presence of the *tetA* gene was significantly associated with tetracycline resistance, with isolates harboring this gene being 5.75 times more likely to exhibit resistance (*p* = 0.016). Similarly, a strong association was observed between the *sul1* gene and resistance to trimethoprim-sulfamethoxazole, with 6.25 times higher odds of resistance in gene-positive isolates (*p* = 0.012). However, no statistically significant association was found between the *strA* gene and streptomycin resistance (OR = 2.04, *p* = 0.203), suggesting that other resistance mechanisms may be involved.

## 4. Discussion

Recent studies have reported a significant increase in *Klebsiella pneumoniae* infections, particularly strains exhibiting multidrug resistance (MDR), including resistance to extended-spectrum β-lactamases (ESBLs), carbapenems, and fluoroquinolones. These resistance patterns complicate treatment regimens, often resulting in prolonged hospital stays and increased morbidity [17,18,32]. The global spread of ESBL- and carbapenemase-producing *K. pneumoniae* has become a pressing public health concern, driven by plasmid-mediated gene transfer and antibiotic misuse in both human and veterinary medicine [33].

In this study, 59 *K. pneumoniae* isolates were identified as MDR, with a notably high resistance to ampicillin (69.9%). This finding indicates a significant prevalence of MDR strains in captive wild and migratory birds [20,34]. Due to ampicillin resistance, up to 90% of *K. pneumoniae* cases in hospitals in northern India occur in patients in the intensive care unit [35]. The presence of MDR *K. pneumoniae* in both wild and captive birds, as well as in poultry environments, underscores the importance of a One Health approach to address antibiotic resistance. This approach considers the interconnectedness of human, animal, and environmental health [18,36].

The prevalence of *K. pneumoniae* in migratory and wild birds varies significantly across different regions, reflecting diverse ecological and environmental factors. In this investigation, 42.47% (93/219) feces samples from captive wild and migratory birds were discovered to have *K. pneumoniae*. This prevalence is lower than the 55.47% reported in China and 53% in Kiel, Germany, but notably higher than the 1.5% in Egypt and comparable to the 20% in Catalonia, Spain [18,22,37,38].

In this investigation, sixty *K. pneumoniae* isolates showed 64.5% (60/93) resistance to aminoglycoside, specifically streptomycin. This resistance may be related to the efficacy of streptomycin treatment for humans and animals in Bangladesh. Combining antibiotics such as streptomycin and penicillin could be a beneficial approach to treat animal infections; however, streptomycin use should be closely monitored [39]. Loss of the KpnO porin and changes in the AcrAb-ToIC and KpnEF efflux pump systems can result in altered cellular permeability, which may lead to *K. pneumoniae* resistance to aminoglycoside drugs [35].

Overuse and incorrect use of antibiotics may lead to antibiotic resistance. Because of the ability of *Klebsiella* to produce the ESBL enzyme, it becomes resistant to antibiotics. Bacterial plasmids may be the source of resistance genes. The gene that produces the ESBL enzyme is based on a plasmid called beta-lactamase, which transforms into a point mutation that modifies the structure of the active region of the original gene [40]. Beta-lactamase enzymes have the ability to shield Gram-negative bacteria from beta-lactamase antibiotics. The cell wall is the target of the beta-lactam antibiotic. Similar to cell walls, this class of antibiotics also contains a beta-lactam group that interacts with enzymes during the production of cell walls. The cell wall will not fully develop since the enzyme will no longer be active. Bacterial cells without cell walls and those with incomplete cell walls result in death [41]. Resistant bacteria can be transferred by pets, wild animals, and food-producing animals [42]. The feces of animals can spread resistant bacteria to nearby people who may have been exposed to the bacteria through the air during animal transportation, as well as to workers in farms and slaughterhouses. These workers may also be infected with resistance bacteria that possess the ability to proliferate the bacteria to the environment [43]. Humans, cattle, wild animals, and non-clinical isolates of *K. pneumoniae* have all been found to produce ESBLs [41,44].

In this investigation, *bla*_TEM_ encoding genes 93/93 (100%) were present in all *K. pneumoniae* positive isolates, whereas *bla*_SHV_ was found in 42 (45.2%) of the 93 positive samples. These results align with those of another study in chickens that found a 100% prevalence of the *bla*_TEM_ gene, reinforcing the high prevalence of this gene in avian sources [45]. In a study involving *K. pneumoniae* isolates from different sources, 84.6% of the isolates were found to carry the *bla*_TEM_ gene, and 73% carried the *bla*_SHV_ gene [46]. This high prevalence of *bla*_TEM_ is consistent with the prevalence reported in this investigation. In addition, the findings are consistent with studies on *Salmonella* spp. from migratory birds, which carried the 94.34% *bla*_TEM_ gene, while 13.33% carried *bla*_SHV_, indicating a similar trend of *bla*_TEM_ dominance [47]. While *bla*_SHV_ was less common than *bla*_TEM_, its presence in a significant portion of isolates indicates that it is also a relevant contributor to beta-lactam resistance in these samples. The existence of the ESBL-coding gene suggests that human resistance genes have migrated to animals, especially the multidrug-resistant *K. pneumoniae*, which is dangerous for livestock and public health [48].

The MAR index, which ranged from 0.18 to 0.64 with an average of 0.33, indicates a significant level of resistance, similar to patterns observed in other studies involving *K. pneumoniae* and other bacteria. This resistance is particularly concerning in environments where antibiotic use is prevalent, such as poultry farms, which can serve as reservoirs for resistant strains that may affect both animals and humans [37]. The presence of MDR isolates, which were resistant to more than three classes of antimicrobials, was observed in 63.4% of the isolates of captive wild and migratory birds, aligning with similar resistance patterns found in various animal and environmental studies [46,47].

Furthermore, this study highlights the prevalence of AMR genes in captive wild and migratory birds, specifically noting the presence of *strA*, *tetA* and *sul1* genes. The study found that 30.1% of isolates were positive for the *strA* gene, which confers resistance to streptomycin. This is consistent with findings from other studies isolated from wild birds in Korea [49]. The *tetA* gene, associated with tetracycline resistance, was found in 9.7% of isolates. This aligns with research that reported a significant presence of tetracycline resistance genes in wild birds in Italy [50]. Other studies have reported similar findings, with *sul1* being prevalent in wild bird populations [47,49]. Wild birds are increasingly recognized as reservoirs and vectors for AMR genes. They can acquire resistant bacteria from contaminated environments and spread them over long distances during migration [51]. The presence of AMR genes in wild birds poses a risk to public health, as these genes can be transferred to human pathogens. This is particularly concerning in areas where human and wildlife habitats overlap [18].

While this study’s findings are interesting, it has several limitations. Environmental samples (e.g., water, soil) were not included, which limits our ability to distinguish between true colonization in birds and potential environmental contamination. The study was also restricted to a specific geographic region and a six months sampling period, which may not capture broader temporal or spatial variations. Additionally, molecular characterization was limited to a few resistance genes, potentially overlooking other important resistance determinants or plasmid types contributing to the observed multidrug resistance.

## 5. Conclusions

This study confirms the presence of *K. pneumoniae* in 42.47% of fecal samples from captive wild and migratory birds in northeastern Bangladesh, with a substantial proportion (63.4%) of isolates exhibiting multidrug resistance. Notably, ß-lactamase encoding genes such as *bla*_TEM-1&2_, *bla*_OXA-1,4&30_, and *bla*_SHV-1_ were detected, indicating a high potential for environmental dissemination of resistance. These findings underscore the role of wild and migratory birds as reservoirs and potential vectors for MDR and ESBL-producing *K. pneumoniae*, posing a threat to public and animal health. Continuous surveillance and prudent antibiotic stewardship under the One Health approach are crucial to mitigate this emerging risk.

## Figures and Tables

**Table 1:** Primer sequence and amplicon size used in this study.

Name of PCR	Primer	Genes Targeted	Primer Sequence (5’–3’)	Amplicon Size	References
Uniplex PCR	*rpoB*	*K. pneumoniae*	F-CAACGGTGTGGTTACTGACG	108	[17]
			R-TCTACGAAGTGGCCGTTTTC		
mPCR-I	*bla*_TEM_	*TEM-1 & 2*	F-CATTTCCGTGTCGCCCTTATTC	800	[25]
			R-CGTTCATCCATAGTTGCCTGAC		
	*bla*_SHV_	*SHV-1*	F-AGCCGCTTGAGCAAATTAAAC	713	
			R-ATCCCGCAGATAAATCACCAC		
	*bla*_OXA_	*OXA-1, 4 & 30*	F-GGCACCAGATTCAACTTTCAAG	564	
			R-GACCCCAAGTTTCCTGTAAGTG		
mPCR-II	*tet (A)*	*tet (A)*	F-GGCGGTCTTCTTCATCATGC	502	[26]
			R-CGGCAGGCAGAGCAAGTAGA		
	*str(A)*	*str(A)*	F-ATGGTGGACCCTAAAACTCT	893	
			R-CGTCTAGGATCGAGACAAAG		
Uniplex PCR	*sul1*	*sul1*	F-CGGCATCGTCAACATAACCT	433	[27]
			R-TGTGCGGATGAAGTCAGCTC		

**Table 2:** Prevalence of *Klebsiella pneumoniae* isolated from captive wild and migratory birds among the different locations in Sylhet Division.

Attributes (Locations)	No. of Isolates Tested	Positive Isolates	Prevalence % (95% CI)	χ^2^ Value	*p*-Value
Sunamganj Haor	94	41	43.6 (33.4–54.2)	7.97	0.047
Haripur	19	12	63.2 (38.4–83.7)		
Sreemangal Zoo	70	31	44.3 (32.4–56.7)		
Tilagor Eco-Park, Sylhet	36	9	25.0 (12.1–42.2)		
Total	219	93	42.47 (35.8–49.3)		

Chi-square (χ^2^) goodness of fit test, CI: Confidence Interval.

**Table 3:** Frequency rate of *K. pneumoniae* isolated from captive wild birds.

Species of Bird	x/N	Frequency %	95% CI	*p*-Value
Dove	2/4	50.0	6.8–93.2	0.48
Hornbill	4/5	80.0	28.4–99.5	
Purple Heron	1/3	33.3	0.8–90.6	
Grey Heron	3/6	50.0	11.8–88.2	
Black Crowned Night Heron	4/6	66.7	22.3–95.7	
Red Jungle Fowl	3/4	75.0	19.4–99.4	
Eagle	2/6	33.3	4.3–77.7	
Mathura	2/3	66.7	9.4–99.2	
Parrot	4/7	57.1	18.4–90.1	
Grey Parrot	3/7	42.9	9.9–81.6	
Peacock	2/5	40.0	5.3–85.3	
Kalim	3/6	50.0	11.8–88.2	
Myna	1/6	16.7	0.4–64.1	
Kite	0/3	0	0–70.8 *	
Vulture	1/3	33.3	0.8–90.6	
Macaw	0/1	0	0–97.5 *	
Golden Pigeon	1/1	100.0	2.5–100.0 *	
Unidentified species	4/4	100.0	39.8–100.0 *	

* Superscript indicates One-sided 97.5% confidence interval.

**Table 4:** Binary logistic regression analysis showing the association between resistance genes and phenotypic antibiotic resistance.

Antibiotic	Resistance Gene	Dependent Variable (Phenotype)	Independent Variable (Genotype)	Odds Ratio (OR)	95% CI (Lower–Upper)	*p*-Value
Tetracycline	*tetA*	TE Resistance (1 = Yes, 0 = No)	Presence of *tetA* (1 = Yes, 0 = No)	5.75	1.4–23.9	0.016
Streptomycin	*strA*	S Resistance (1 = Yes, 0 = No)	Presence of *strA* (1 = Yes, 0 = No)	2.04	0.68–6.2	0.203
Trimethoprim-Sulfamethoxazole	*sul1*	SXT Resistance (1 = Yes, 0 = No)	Presence of *sul1* (1 = Yes, 0 = No)	6.25	1.5–26.2	0.012

## Data Availability

All data generated and analyzed in this study are included in the main manuscript.

## Supplementary Materials

The following supporting information can be downloaded at: https://www.mdpi.com/article/10.3390/vetsci12060556/s1; **Figure S1.** (**A**–**E**): Electrophoresis on 1.5% agarose gel showing specific amplified band of different gene of *Klebsiella pneumoniae*. amplification by PCR; Lane M: 100 bp Marker DNA; Lane NC: Control (– v e); **A**: Lane (1-10) reaction specific (+ ve) for *Klebsiella pneumoniae* (108 bp); **B**: Lane (1-10) samples for ESBL genes (*bla_TEM-1 & 2_* at 800 bp, *bla_SHV-1_* at 713, *bla_OXA-1,4 & 30_* at 564); **C**: Lane 1-7 samples for *strA* gene (893 bp); **D**: Lane 1-5 for *tetA* gene (502 bp); **E**: Lane (1-5) samples for *sul1* gene (433 bp); **Table S1**: List of Antibiotic disks for antimicrobial susceptibility testing; **Table S2:** Antibiogram profile of *K. pneumoniae.*
