# Supplementary material for: Molecular Identification and Antimicrobial Resistance Characteristics of Extended-Spectrum Beta-Lactamase Producing Klebsiella pneumoniae Isolated from Captive Wild and Migratory Birds
Source: Vet Sci. 2025 Jun 6;12(6):556. doi: 10.3390/vetsci12060556 (PMC12197686; doi:10.3390/vetsci12060556)
Supplement: Supplementary file 1 [file vetsci-12-00556-s001.zip › vetsci-3628180-supplementary.pdf]

Supplementary Table S1: List of Antibiotic disks for antimicrobial susceptibility testing

**Sl. No.**	**Drug class or subclass**	**Antimicrobial agents**	**Abbreviations**	**Disk content**	**Inhibition zone (mm)**		
					**S**	**I**	**R**
1	Penicillins	Ampicillin	AMP	10 µg	≥ 17	14-16	≤ 13
2	Aminoglycosides	Gentamicin	CN	10 µg	≥ 15	13-14	≤ 12
		Streptomycin	S	10 µg	≥ 15	12-14	≤ 11
3	Cephalosporins	Cefixime	CFM	5 µg	≥ 19	16-18	≤ 15
		Ceftriaxone	CRO	30 µg	≥ 23	20-22	≤ 19
4	Carbapenem	Meropenem	MEM	10 µg	≥ 23	20-22	≤ 19
5	Tetracyclines	Tetracycline	TE	30 µg	≥15	12-14	≤ 11
6	Fluoroquinolones	Ciprofloxacin	CIP	5 µg	≥ 26	22-25	≤ 21
		Levofloxacin	LEV	5 µg	≥ 21	17-20	≤ 16
8	Macrolids	Azithromycin	AZM	15 µg	≥ 13	-	≤ 12
9	Sulfonamides	Trimethoprim-Sulfamethoxazole	SXT	1.25/ 23.75 µg	≥ 16	11-15	≤ 10

S= Sensitive, I= Intermediate, R= Resistant

Supplementary Table S2: Antibiogram profile of *K. pneumoniae*

Antibiotic disk	Resistant (%)	Intermediate (%)	Sensitive (%)
AMP	69.89	24.73	5.38
CN	9.68	20.43	69.89
S	64.5	0	35.5
CFM	24.73	10.75	64.52
CRO	20.43	24.73	54.84
MEM	15.05	24.73	60.22
TE	19.36	5.38	75.26
CIP	9.68	35.48	54.84
LEV	10.75	9.68	79.57
AZM	60	0	40
SXT	15.05	0	84.95

Results of different Biochemical tests for *Klebsiella* isolates

SL. No	Name of Tests	Results	Interpretation
1	Sugar Fermentation test by TSI (Triple Sugar Iron) Agar	Yellow color slant and butt	TSI (+)ve
2	Methyl Red test	Yellow color	Methyl Red (-)ve
3	Citrate Utilization Test by Simmon's Citrate Agar	Bright blue	Citrate(+)ve

Thermal cycle for mPCR of blaTEM, blaSHV and blaOXA

Sl no.	Steps	Temperature (°C)	Time	Cycle	References
1	Initial denaturation	95°C	5 min	1	Bobbadi et al.,2020)
2	Final denaturation	94°C	40 sec	30	
3	Annealing	60°C	40 sec		
4	Initial Extension	72°C	1 min		
5	Final Extension	72°C	7 min	1	

Thermal cycle for Tetracycline resistance gene (tet A) and Streptomycin resistance gene (str A)

Sl no.	Steps	Temperature (°C)	Time	Cycle	References
1	Initial denaturation	94°C	15 min	1	Bobbadi et al.,2020)
2	Final denaturation	94°C	1 min	30	
3	Annealing	63°C	1 min		
4	Initial Extension	72°C	1 min		
5	Final Extension	72°C	10 min	1	
