# Supplementary figures and images for: Molecular Identification and Antimicrobial Resistance Characteristics of Extended-Spectrum Beta-Lactamase Producing Klebsiella pneumoniae Isolated from Captive Wild and Migratory Birds
Source: Vet Sci. 2025 Jun 6;12(6):556. doi: 10.3390/vetsci12060556 (PMC12197686; doi:10.3390/vetsci12060556)

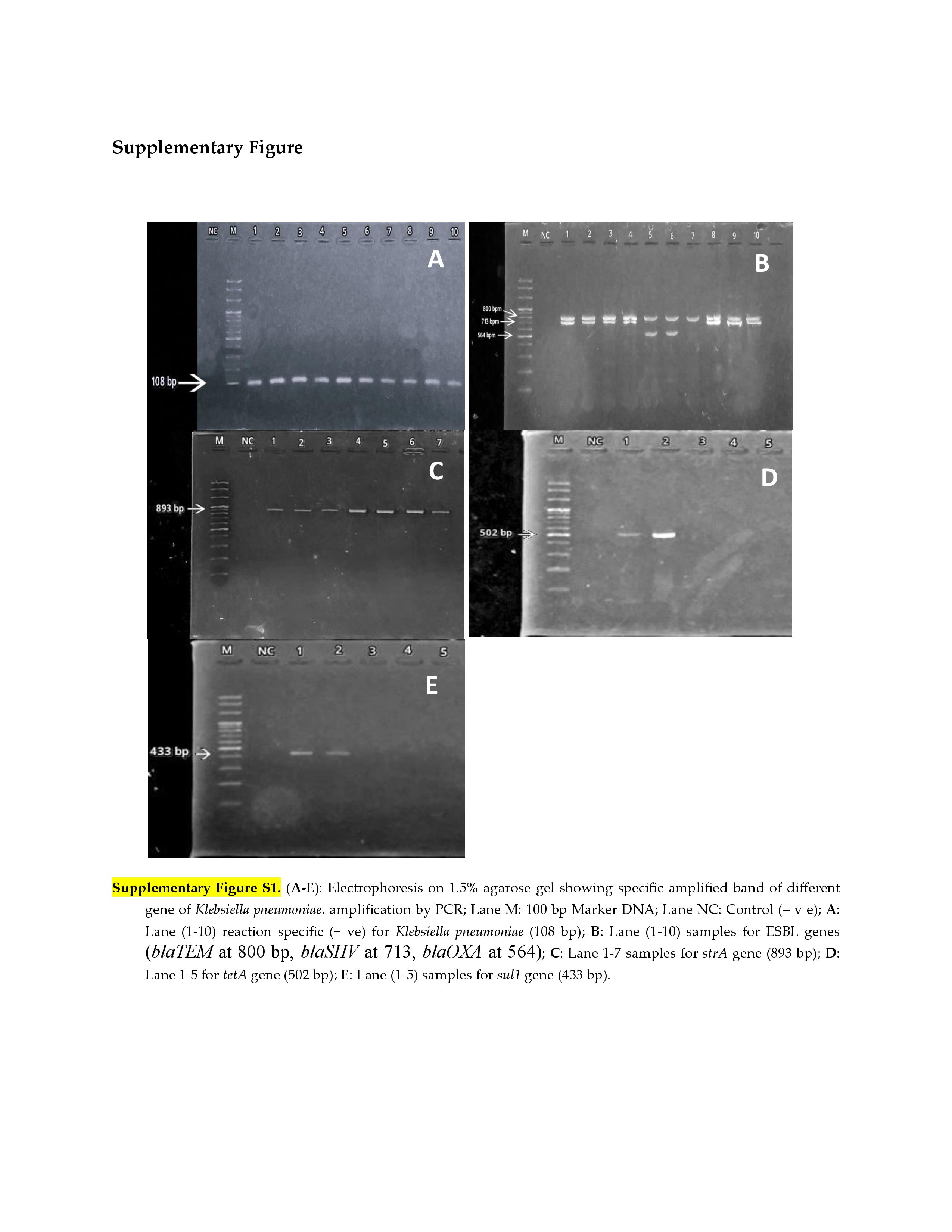

Supplement: Supplementary file 1 [file vetsci-12-00556-s001.zip › vetsci-3628180-figures.jpg]
